# Biology Needs Evolutionary Software Tools: Let’s Build Them Right

**DOI:** 10.1093/molbev/msy084

**Published:** 2018-04-23

**Authors:** Anton Nekrutenko, Galaxy Team, Jeremy Goecks, James Taylor, Daniel Blankenberg

**Affiliations:** 1 Department of Biochemistry and Molecular Biology, The Pennsylvania State University, University Park, PA; 2 The Galaxy Project, https://github.com/orgs/galaxyproject/people Biomedical Engineering; 3 Department, Computational Biology Program, Oregon Health and Science University, Portland, OR; 4 Department of Biology, Johns Hopkins University, Baltimore, MD; 5 Lerner Research Institute, Cleveland Clinic, Cleveland, OH

**Keywords:** software, evolutionary biology, computational biology

## Abstract

Research in population genetics and evolutionary biology has always provided a computational backbone for life sciences as a whole. Today evolutionary and population biology reasoning are essential for interpretation of large complex datasets that are characteristic of all domains of today’s life sciences ranging from cancer biology to microbial ecology. This situation makes algorithms and software tools developed by our community more important than ever before. This means that we, developers of software tool for molecular evolutionary analyses, now have a shared responsibility to make these tools accessible using modern technological developments as well as provide adequate documentation and training.

When Lewontin and Hubby (Hubby and Lewontin 1966; Lewontin and Hubby 1966) demonstrated that genetic variation in natural populations can be observed directly at high resolution (i.e., protein-level), they provided evolutionary and population biology with the ability to generate much more interesting and insightful datasets. Initially, these datasets were small by today’s standards. For example, their two classical *Genetics* papers contained all (!) data in the main text along with all calculations. The development of recombinant DNA and sequence determination techniques in the 1970s allowed for generation of larger datasets such as the sequencing of an entire alcohol dehydrogenase gene from several populations of *Drosophila* in early 1980s (Kreitman 1983). That same period of late 1970s and early 1980s also saw the emergence of personal computers and the development of the first evolutionary analysis toolkit—PHYLIP (Felsenstein 1993)—the oldest continuously maintained software in our field. In mid-2000 the development of next-generation sequencing techniques has brought low-cost, high output data generation capacity to all areas of life sciences. A by-product of this data explosion was that a number of biomedical domains that were traditionally distant from evolutionary thinking found themselves in a situation where data interpretation should be performed in the evolutionary context. For example, analyses of infectious diseases such as AIDS and influenza, proliferation of malignant tumors, emergence of antibiotic resistance, and many others types of problems can only be fully understood in the context of evolutionary analyses. Truly, Dobzhansky (1973) was right in saying that “Nothing in biology makes sense except in the light of evolution”. These unique circumstances position evolutionary and population biology at the center of life sciences—a place well deserved. But it also places a special responsibility on us—practitioners of this field—to make our software tools useful and comprehensible by the broad life sciences community. Below, we examine recent developments that would make this possible.

To be usable, software tools should minimally be accessible and (well) documented. To gauge these parameters within recently published molecular evolution software tools we have examined all *Methods* and *Resource* articles published in *MBE* between January 2017 and March 2018. We only looked at articles that were either freely available (outside the paywall) or had a clearly specified URL pointing to the software within the abstract. This is because readers from other biological domains are unlikely to be subscribed to *MBE*. There were 23 papers describing new software tools (see supplementary table S1). Twenty-two had source code deposited in GitHub or R archive (The Comprehensive R Archive Network [CRAN])—a testament to the openness of the field. We then looked at how easily these tools can be used in practice. This presented a less exciting picture: only three tools contained enough information (documentation and/or tutorials) to actually be easily installed and used. Two additional tools has been added to Bioconda (see below) greatly improving their usability. Thus the conclusion so far is that the community is open (the absolute majority of tools are in the open source domain) but significantly lacks in the area of making tools truly usable. Below we summarize technological developments that can significantly improve the usability of our software while putting minimal strain on developers. Specifically, we discuss advances in package and environment management for installing tools, software containerization for isolating tools and dependencies, and integrative frameworks that provide access to a wide range of tools through a single user interface (UI) (fig. 1).

**Fig. 1.**
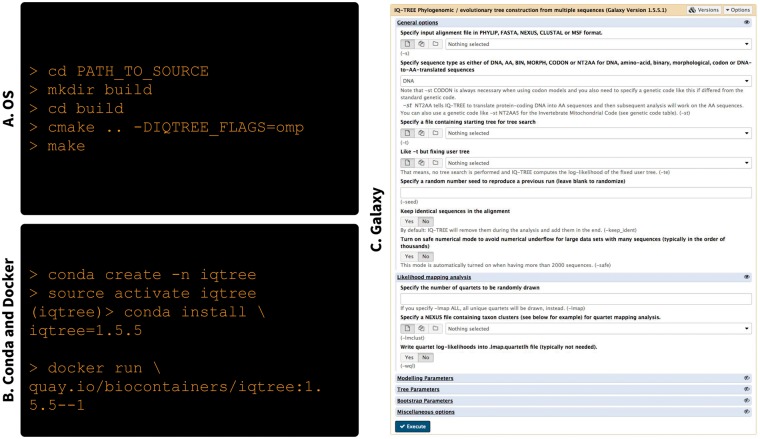
Examples of different deployment strategies for a single tool IQ-Tree (Nguyen et al. 2015). (*A*) Compiling from the source code on Linux (installation instruction specific to MacOS and Windows are described in IQ-Tree website). These instruction do not include installation of compiler and cmake as well as environment configuration (e.g., PATH variable). (*B*) Because IQ-Tree is available from Bioconda (https://bioconda.github.io/recipes/iqtree, last accessed March 2018) is can be installed with much less effort. Here we first create an isolated virtual environment (conda create), switch to that environment (source activate), and finally install IQ-Tree itself (conda install). In contrast to (*A*) this takes care of all dependencies and environment configuration making the package immediately ready for use. Because Bioconda automatically creates containers the tool can be run from within a container (docker run). Note that in these cases (Conda and Docker) we explicitly specify version of IQ-Tree (1.5.5). Ability of specify software versions is essential for making analyses transparent and reproducible. (*C*) Finally, because IQ-Tree is already in Conda it is trivial to incorporate it into Galaxy—an integrative environment (this screenshot is from http://usegalaxy.eu, last accessed March 2018). This provides users with a consistent interface and ability to combine IQ-Tree with other tools within a Galaxy such as, for example, tools for generation of multiple alignments.

## Tools for Packaging and Distributing Software

Several recent developments promise to significantly help make software distribution less of a burden on developers. The first of these developments is evolution of frameworks for management of tool dependencies and runtime environments. The biggest problem for many (especially naïve) users is installation, especially when the tool needs to be compiled from the source code and properly installed (fig. 1*A*). The situation is often aggravated by dependencies such as external libraries required for successful building of executables within a multitude of operating systems (OSs) and local configurations. Conda (https://conda.io, last accessed March 2018) represents the latest generation of open source package and environment managers developed specifically to mitigate this issue (fig. 1*B*). With Conda, tools and their dependencies can be easily installed through a simple, one-step command. Conda also works across programming languages and OSs, making it widely useful. Leveraging Conda, Bioconda (https://bioconda.github.io, last accessed March 2018) is a community project dedicated to data analysis in life sciences that contains over 3,700 tool packages with contributions by more than 400 authors (Dale et al. 2017). Despite the fact that Bioconda is one of the most recent package managers dedicated to biomedical tools, it contains by far the largest number of software tools, underscoring its rapid uptake by the community (fig. 2 in Dale et al. 2017). Bioconda packages are well maintained and include a testing system to ensure their quality.

Another transformative development is software containerization platforms (or, simply, containers) represented by Docker (https://www.docker.com, last accessed March 2018), Singularity (Kurtzer et al. 2017), and rkt (https://coreos.com/rkt, last accessed March 2018). Containers are run within host’s OS’s kernel but “containerize” every other aspect of the runtime environment, providing higher isolation from local environment compared with what Conda virtual environments can provide (these can still be influenced by the host system; Beaulieu-Jones and Greene 2017). Containers share a kernel with the host environment, thus the impact on execution performance is negligible while enabling computational reproducibility akin to full virtual machines. Contains are straightforward to create and they are automatically generated for every tool included into Bioconda (Dale et al. 2017).

## Integrative Frameworks

Integrative frameworks are systems where heterogeneous tools can be applied to a variety of datasets within a single, unified UI. There are many advantages to such systems: they provide users with ready-to-use tools that can be combined into complete workflows, support data storage and computational needs, automatically convert between file formats, and provide capabilities for reproducing and sharing analyses. Galaxy (Blankenberg et al. 2007; Goecks et al. 2010; Afgan et al. 2016) is the most widely used of these platforms (http://bit.ly/gxyTSstats, last accessed March 2018). It provides access to hundreds of tools used in a wide variety of analysis scenarios (e.g., through American http://usegalaxy.org [last accessed March 2018], European http://usegalaxy.eu [last accessed March 2018], and Australian http://usegalaxy.org.au [last accessed March 2018] server instances). It features a web-based UI while automatically and transparently managing underlying computation details. In addition to public servers, it can be deployed on a personal computer, heterogeneous computer clusters, as well as computation systems provided by Amazon, Microsoft, Google, and other clouds. It is an open, community driven project, which ensures its sustainability and allows it to be adapted for use in a wide variety of research domains from genomics to image analysis to natural language processing.

The advantage of integrative frameworks is that they provide multiple tools under the umbrella of a single system. This means that a user can perform complex, multi-step analyses in one place. For example, a researcher studying evolution of antibiotic resistance can start from the very beginning by assessing the quality and mapping of, say, Illumina data, calling and filtering variants, and identifying sites under selection all within one system without ever leaving it and never needing to install anything. One can argue that such a statement—performing everything within a single system—is unrealistic because 1) one cannot always assess the full complexity of a given analysis *a priori* and 2) systems like Galaxy can never include all possible tools. This is true and this is why we have developed Interactive Environments (IEs) within Galaxy (Grüning et al. 2016). Using IEs, one can start a Jupyter and RStudio session directly within Galaxy (using its robust computational infrastructure) and perform any type of *ad hoc* analysis such as a statistical test or creating a custom visualization.

Any web-based or command-line tools can be integrated into Galaxy. However, tools that are already registered with Conda (or Bioconda) are especially easy to add because all dependency resolution issues are already solved by the package manager or software containers (fig. 1*C*). Depending on its configuration, Galaxy can create a dedicated Conda environment for tool execution or “pull” (download) a Docker container that was automatically created by Bioconda and invoke the tool within this container.

## Training Is Key

Expansion of areas that can directly benefit from software tools developed within evolutionary biology means that numerous researchers unfamiliar with these types of analyses will need to be trained. This means that 1) a framework for distribution and management of educational materials should be developed and 2) community-sourced tutorials need to be produced.

To achieve the first goal, we and the Galaxy community have built an infrastructure for creation and delivery of training materials that enables transparent peer-review and curation to guarantee high quality and current content. In doing this we took inspiration from the Software and Data Carpentry (SDC) (Wilson 2014) projects where materials are openly reviewed and iteratively developed on GitHub (https://github.com/, last accessed March 2018) to capture the breadth of community expertise. SDC delivers training via online tutorials with hands-on sections, which offer better training support than videos because trainees who are actively participating learn more (Dollar et al. 2007). The content of these web pages is easy to edit, thus reducing the contribution barrier. The tutorials are developed in Markdown, a plain text markup language, which is automatically transformed into web-browser accessible pages. Using these strategies, we created a GitHub repository (https://github.com/galaxyproject/training-material, last accessed March 2018) to collect, manage, and distribute training materials. This infrastructure has been developed in accordance with the FAIR (Findable, Accessible, Interoperable, Reusable) principles (Wilkinson et al. 2016). Using the framework described above, we relaunched the Galaxy Training Network (GTN; https://galaxyproject.org/teach/gtn, last accessed March 2018). This growing network currently consists of 33 scientific groups (https://galaxyproject.org/teach/trainers, last accessed March 2018) invested in Galaxy-based training. The GTN regularly organizes training events worldwide and offers best practices for developing Galaxy-based training material, advice on compute platform choice to use for training, and a catalog of existing training resources for Galaxy. There is currently a paucity of tutorials targeting evolutionary- and population-biology types of analyses. We hope that this report will precipitate their development.

## Going Forward

Concluding, we would like to introduce a short set of recommendations that can potentially widen the impact of the software produced within the field of evolutionary biology.

1. **Use modern software distribution practices**. Using systems like Conda dramatically simplifies installation of software tools for end users. The importance of this cannot be overemphasized. Many readers will recall “horrors” of source code not compiling properly or searching for the right version of a needed software library. For a naïve user such a situation is the end of an attempt to ever try the software. Using Conda reduces this unnecessary complexity to simply using conda install, which will automatically retrieve dependencies and install needed components. This does not only benefit the user. This benefits the software developer as well. After all, the “fitness” of software is directly proportional to the number of users and these approaches will increase the number of users.
2. **Use integrative environments** because stand-alone web applications have limited utility. It is often tempting to develop a web-server for a singular tool or a collection of tools. However single-purpose web servers usually do not have all tools necessary for performing a complete from-data-to-publication type of analysis. For example, a website implementing a tree reconstruction algorithm (such as PhyML; Guindon et al. 2010) will use sequence alignments in a particular format (e.g., Newick) as the input. But these alignments need to be generated somehow and converted to an appropriate format—a set of manipulations the website is unlikely to provide. On the other hand, incorporating the tool into a system like Galaxy empowers users to combine the tool in novel ways with hundreds of other utilities as well as to interactive computing environments such as Jupyter and RStudio. This also frees developers from website development—significant time that can be spent, well, wrapping tools in Conda, Galaxy, and developing tutorials.
3. **Documentation and training efforts always pay off.** It is redundant to say that documentation is key to everything. Tutorial development is hard work because one needs to design analyses using specially tailored minimal datasets that will produce meaningful results and tell an engaging story. However, only domain specialists can produce quality educational materials and so we appeal to all readers of this piece: if you have ever developed an analysis tool, make a tutorial to showcase what your tool can do. Ultimately (as we mentioned above) this will only increase the “fitness” of your software.

## Supplementary Material

Supplementary data are available at *Molecular Biology and Evolution* online.

## Supplementary Material

Supplementary Data
