# Supplementary material for: Biology Needs Evolutionary Software Tools: Let’s Build Them Right
Source: Mol Biol Evol. 2018 Apr 23;35(6):1372–5. doi: 10.1093/molbev/msy084 (PMC5967460; doi:10.1093/molbev/msy084)
Supplement: Supplementary Data [file msy084_supp.pdf]

Paper	Source	Is in Conda?	Notes	Resource Name	Resource Link	Web or Desktop
https://doi.org/10.1093/molbev/msw210	http://cran.R-project.org	no	Broken link in the paper	haploReconstruct: Re	https://cran.r-project.org/	Desktop
https://doi.org/10.1093/molbev/msw217	github	no		PANGEA Phylodynam	https://github.com/Pange	Desktop
https://doi.org/10.1093/molbev/msw220	github	no	Broken link in the paper	PhyloBayes-MPI	https://github.com/bayes	Desktop
https://doi.org/10.1093/molbev/msw227	github	no		CTDGFinder	https://github.com/biofilc	Desktop
https://doi.org/10.1093/molbev/msw211	github	no	very nice site	SLiM	https://messerlab.org/slin	Desktop
https://doi.org/10.1093/molbev/msw252	github	no	bad readme, figure links broken on MBE website	IMGui	https://github.com/jaredg	Desktop
https://doi.org/10.1093/molbev/msw267		no	not really a tool paper			
https://doi.org/10.1093/molbev/msw260	github	no	very nice manual	PartitionFinder 2	http://www.robertlanfear	Desktop
https://doi.org/10.1093/molbev/msw275	github	no	R package, very nice manual	TransPhylo	https://github.com/xavier	Desktop
https://doi.org/10.1093/molbev/msx055	github	no	tool comparison, github contains datasets and scripts		https://github.com/stamatak/tree-viz-issues	
https://doi.org/10.1093/molbev/msx089	pip/github	no		BuddySuite	https://research.nhgri.nih	Desktop
https://doi.org/10.1093/molbev/msx116	unclear	no	Confusing disclaimer	TimeTree	http://www.timetree.org/	Web
https://doi.org/10.1093/molbev/msx124	github	no	data is at github	genPomp	https://github.com/kinga	Desktop
https://doi.org/10.1093/molbev/msx137	https://sites.google.com/site/cbic	no	source download link dead	ETDBN	http://www.computingfor	Desktop
https://doi.org/10.1093/molbev/msx126	github	no		StarBEAST2	https://github.com/genon	Desktop
https://doi.org/10.1093/molbev/msx148	github / website	no		eggNOG-Mapper	http://eggnogdb.embl.de/	Both
https://doi.org/10.1093/molbev/msx191	github	no		Bad Clade Deletion (E	https://bio.informatik.uni	Desktop
https://doi.org/10.1093/molbev/msx149	PhyML	yes		Smart Model Selectio	http://www.atgc-montpel	Both
https://doi.org/10.1093/molbev/msx200	github	yes	a new method by combining other methods, no	Ray Surveyor	https://github.com/zorinc	Desktop
https://doi.org/10.1093/molbev/msx186	github	no		The Structured Coale	https://github.com/nicfel/	Desktop
https://doi.org/10.1093/molbev/msx195	github	no		EpiGenMCMC	https://github.com/lucym	Desktop
https://doi.org/10.1093/molbev/msx209	github	no		BetaScan	https://github.com/ksiew	Desktop
https://doi.org/10.1093/molbev/msx225	github/R	no		poolSeq	https://github.com/Thom	Desktop
https://doi.org/10.1093/molbev/msx259	github	no		STRIDE	https://github.com/david	Desktop
https://doi.org/10.1093/molbev/msx235	github	no		PhyloNetworks	https://github.com/crsl4/I	Desktop
